# Predicting Lung Cancer Survival Using Probabilistic Reclassification of TNM Editions With a Bayesian Network

**DOI:** 10.1200/CCI.19.00136

**Published:** 2020-05-11

**Authors:** Melle S. Sieswerda, Inigo Bermejo, Gijs Geleijnse, Mieke J. Aarts, Valery E.P.P. Lemmens, Dirk De Ruysscher, André L.A.J. Dekker, Xander A.A.M Verbeek

**Affiliations:** ^1^ Department of Research and Development, Netherlands Comprehensive Cancer Organization, Utrecht, the Netherlands; ^2^ Department of Radiation Oncology (MAASTRO), GROW School for Oncology and Developmental Biology, Maastricht University Medical Centre, Maastricht, the Netherlands

## Abstract

**PURPOSE:**

The TNM classification system is used for prognosis, treatment, and research. Regular updates potentially break backward compatibility. Reclassification is not always possible, is labor intensive, or requires additional data. We developed a Bayesian network (BN) for reclassifying the 5th, 6th, and 7th editions of the TNM and predicting survival for non–small-cell lung cancer (NSCLC) without training data with known classifications in multiple editions.

**METHODS:**

Data were obtained from the Netherlands Cancer Registry (n = 146,084). A BN was designed with nodes for TNM edition and survival, and a group of nodes was designed for all TNM editions, with a group for edition 7 only. Before learning conditional probabilities, priors for relations between the groups were manually specified after analysis of changes between editions. For performance evaluation only, part of the 7th edition test data were manually reclassified. Performance was evaluated using sensitivity, specificity, and accuracy. Two-year survival was evaluated with the receiver operating characteristic area under the curve (AUC), and model calibration was visualized.

**RESULTS:**

Manual reclassification of 7th to 6th edition stage group as ground truth for testing was impossible in 5.6% of the patients. Predicting 6th edition stage grouping using 7th edition data and vice versa resulted in average accuracies, sensitivities, and specificities between 0.85 and 0.99. The AUC for 2-year survival was 0.81.

**CONCLUSION:**

We have successfully created a BN for reclassifying TNM stage grouping across TNM editions and predicting survival in NSCLC without knowing the true TNM classification in various editions in the training set. We suggest binary prediction of survival is less relevant than predicted probability and model calibration. For research, probabilities can be used for weighted reclassification.

## INTRODUCTION

In cancer care, the TNM system for classification of malignant tumors guides treatment decisions, aids in stratifying patients for research, and helps clinicians assess prognosis.^1,2^ This is done by classifying characteristics of the tumor (T descriptor), local lymph nodes (N descriptor), and distant metastases (M descriptor). These descriptors can subsequently be used to compute a stage grouping, essentially summarizing the information.

**CONTEXT**

**Key Objective**
The TNM classification system is used for prognosis, treatment, and research; however, data classified with different editions are not directly comparable. Reclassifying data across TNM editions would facilitate pooling historical data for research.
**Knowledge Generated**
Bayesian networks can be used to reclassify stage grouping across TNM editions by combining clinical knowledge with real-world data and leveraging the relation between TNM stage and survival.
**Relevance**
Probabilistic reclassification across TNM editions for NSLCL makes it possible to use data classified with different TNM editions for research. Secondly, our Bayesian network offers multiclass survival prediction with accompanying probabilities, possibly helping to assess prognosis.

The system is revised on a regular basis (every 5.7 years on average), during which changes are made both to the individual descriptors and to the stage grouping.^3^ Revisions incorporate new developments that improve (outcome) stratification and prognostic capabilities, keeping the classification system relevant. However, because categories can be added or removed, classes with the same label are not necessarily equivalent across editions.^4^ Recommendations for care interventions from the literature and clinical trials may be based on specific editions of the classification system, and it is not always immediately clear how to apply these recommendations to patients classified with a different edition.^5^ Also, scientific analysis of patient cohorts classified with different editions must consider the differences across editions.

This issue can be tackled by either mapping class labels from source to target edition or by (re)classifying the patient using the target edition. If mapping is not feasible, additional data are required to help determine the individual descriptors in the target edition. In practice, this process is complicated, and these data are usually excluded from the analyses or an approximate mapping is assumed.

For both situations described, it would be helpful to have a model that can aid in the reclassification across TNM editions. In this article, we develop such a model based on Bayesian networks (BNs).

A BN is a type of probabilistic graphic model that uses a directed acyclic graph. The nodes represent variables, and the directed edges signify (preferably causal) relationships.^6,7^ Each node is associated with a probability distribution that is conditional on its parents (ie, the set of nodes that have a directed edge to that node), which can be written as $P\left(X\text{|}Pa_{X}\right)$. This leads to a set of (conditional) probability distributions that together define a joint probability distribution, which can be written as $P\left(X_{1},\;\ldots ,\;X_{n}\right)=\overset{n}{\underset{i=0}{\prod }}P\left(X_{i}\text{|}Pa_{X_{i}}\right)$. For nodes that are associated with a discrete probability distribution, the distribution is defined as a conditional probability table (CPT).

BNs can be used to estimate the probability distribution of a variable given evidence. In contrast to other models, such as logistic regression, BNs do not have dedicated inputs or outputs. Instead, setting evidence on any node updates probabilities throughout the network.

The resulting models can easily be evaluated by medical specialists: the graphic nature of BNs makes interpretation of relationships straightforward, and conditional probability aligns well with physicians’ reasoning. This is a benefit over black-box approaches, such as artificial neural networks or deep learning.

In this work, we hypothesized BNs can be used to reclassify data across TNM editions for non–small-cell lung cancer (NSCLC) and predict survival. We further hypothesized that such a BN can be learned without knowing the true TNM classification in various editions in the training set by leveraging the correlation between TNM and survival.

## METHODS

### Data

Data were obtained from the population-based Netherlands Cancer Registry (NCR) after approval by the NCR Privacy Review Board and did not require approval from an ethics committee in the Netherlands. The NCR has been maintained since 1989 and is populated by trained data managers. It contains all cancer occurrences in the Netherlands. Coding is based on international rules and standards. The edition of the TNM classification used depends on the incidence year (Table 1).

**TABLE 1.**
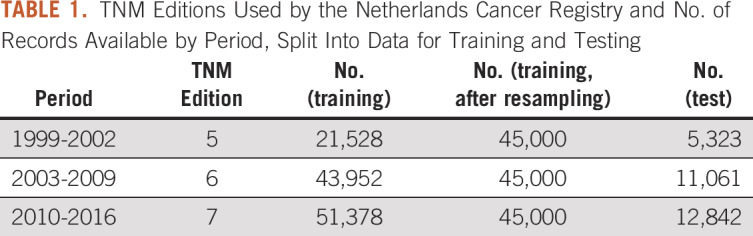
TNM Editions Used by the Netherlands Cancer Registry and No. of Records Available by Period, Split Into Data for Training and Testing

Inclusion criteria were pathology confirmed, International Classification of Diseases *O*-3 topology code C34 (bronchus and lung), morphology codes appropriate for NSCLC, and incidence year between 1999 and 2016; 2017 and 2018 were excluded because of insufficient follow-up. Patients with multiple primary tumors were kept because lung cancer is generally dominant in determining survival. A total of 146,084 patients fulfilled our criteria. We obtained the following variables: clinically staged T, N, and M descriptors; incidence year; days of follow-up; and vital status at follow-up. A new variable was added to each record specifying the TNM edition used, based on year of incidence. Survival time, counted from initial diagnosis, was discretized into 5 categories frequently used in the literature and clinical practice (Table 2). Discretization introduced missing values for patients who had a follow-up of less than 2 years and were alive at the time of follow-up. The dataset was randomly split into a training set (80%; n = 116,858) and a test set (20%; n = 29,226). Because the distribution of the different TNM editions was unequal, the training set was resampled (with replacement) to contain 45,000 samples for each edition. NAs were removed from the test set.

**TABLE 2.**
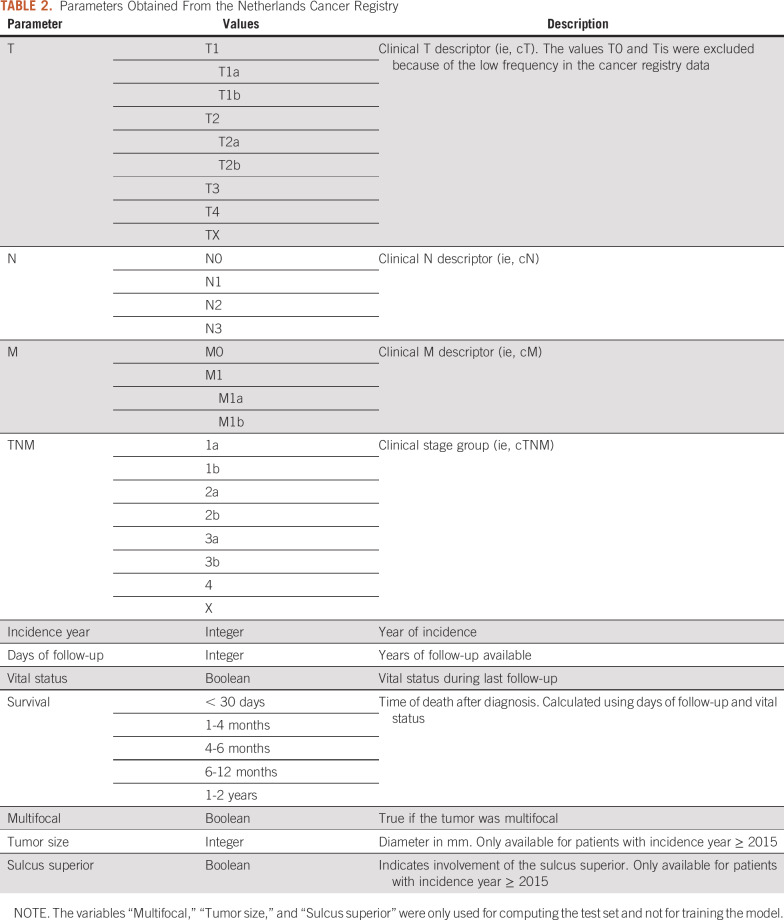
Parameters Obtained From the Netherlands Cancer Registry

### Network Structure Definition

**FIG 1.**
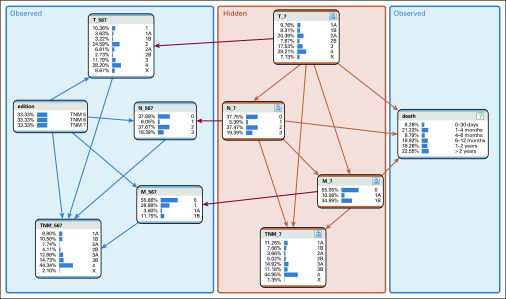
Bayesian network (after Expectation-Maximization learning) visualized using BayesiaLab 8. Each node represents a random variable with a (conditional) probability table and shows its state’s prior probabilities in a (rotated) histogram. The arrows indicate the (causal) relations between the nodes. The “_567”-suffix indicate nodes that, in conjunction with the “edition” node, can take on values from all TNM editions. The “_7”-suffixed nodes can take on 7th edition values only.

A BN was designed to predict the 5th and 6th TNM editions with variables from the 7th edition of the TNM classification (TNM7) and vice versa, shown in Figure 1, using BayesiaLab 8.^8^ This required 2 sets of 4 nodes corresponding to T, N, and M descriptors and TNM stage grouping: one set for input and one for output. By adding 2 nodes for TNM edition and survival, a total of 10 nodes was obtained. Relationships between nodes were established to indicate causal effect. Nodes edition, T_567, N_567, M_567, TNM_567, and death were associated with variables in the dataset, leaving T_7, N_7, M_7, and TNM_7 as hidden nodes. CPTs for the hidden nodes were estimated on a subset of the training set containing only 7th edition data. Relationships between hidden nodes and their observed counterparts were primed by manually estimating CPTs P(T_567|edition,T_7),P(N_567|edition,N_7), and P(M_567|edition,M_7) through analysis of the differences between editions 6 and 7 in the American Joint Committee on Cancer (AJCC) staging manual (Data Supplement); there were no changes between editions 5 and 6, and survival differed only marginally (Data Supplement). Finally, Expectation-Maximization learning was used to estimate the CPTs of the full network.^9^

### Reclassifying TNM7 Data as Ground Truth for Testing

Evaluating network reclassification performance requires a ground truth. Therefore, a subset of the test set was manually reclassified as follows.

The additional parameters of multifocal disease, tumor size, and sulcus superior involvement were obtained from the NCR for patients in the dataset (Table 2). Because collection of tumor size and sulcus superior involvement started in 2015, the subset was limited to include patients diagnosed in 2015 and 2016 (n = 3,544).

By comparing definitions for the T, N, and M descriptors in the AJCC staging manuals, reclassification rules were defined (Data Supplement). If a one-to-one mapping was impossible, determining a range of values was attempted. With the individually reclassified descriptors, the TNM stage group was computed.

### Evaluating Predictive Network Performance

The network’s performance was evaluated on (1) predicting the 6th edition stage grouping, (2) predicting the 7th edition stage grouping using the ground truth set, and (3) predicting survival using the test set. Predicting the 5th edition was not evaluated separately, because the 5th and 6th editions are equivalent. The HUGIN Analysis Wizard was used to compute the confusion matrix for each evaluation by selecting the state with the highest belief as predicted state.

Macro-averaging and micro-averaging are used in multiclass problems to combine multiple metrics into single values (Data Supplement).^10^ Micro-averaged and macro-averaged sensitivity, specificity, and accuracy were computed with the Python programming language.^11-14^ We additionally used BayesiaLab to calculate the receiver operating statistic area under the curve (ROC-AUC) for 2-year survival.

Model calibration for survival was visualized using a bubble plot and curve. Briefly, each unique combination of inputs (ie, edition T_567, N_567, M_567, and TNM_567) defines a subpopulation. The plots show, for each subpopulation, the relation between a survival category’s predicted probability and its observed frequency in the dataset. The size of each bubble indicates the population’s size in the dataset. The calibration curve, computed using scikit-learn in Python, averages predicted and observed values by applying quantile-based binning with 1,000 samples per bin (21 bins).^14,15^

## RESULTS

### Network Structure and Parameters

The final network structure is shown in Figure 1. Each node represents a random variable and shows its state’s prior probabilities in a histogram. The arrows indicate (causal) relations between the nodes. A node’s underlying CPT is conditional on its parents.

We assumed that the newest available TNM edition approximates the true disease state best and thus has a causal relation with survival. This also means that because the TNM edition used for staging a patient should not (causally) influence survival, each of the nodes T_567, N_567, and M_567 has 2 parents: edition and its corresponding unobserved/hidden counterpart.

After the network structure was created, Expectation-Maximization learning was successfully applied. Percentages shown on each node show the prior probabilities for each state. In general, the model behaved as expected: an increase in tumor stage in any of the T, N, M, and TNM nodes corresponded to poorer survival. Additionally, the relations between the hidden and the observed TNM nodes were close to the priors set manually.

### Manual Reclassification of the Dataset

Multifocality, tumor size, and sulcus superior involvement were not always available for all patients. For 150 records (4%), the T descriptor could not be determined because of these missing values. A total of 451 patients (13%) had multifocal T4 tumors according to the 7th edition for which it was not possible to determine the corresponding 6th edition T descriptor without additional information. Another 216 records (6%) could only be classified into a range (eg, T2-T3). As a result, for 817 records (23%), the T descriptor could not be completely reclassified (Data Supplement). The N descriptor could be reclassified in all patients. There were 85 multifocal T4 tumors (2%) that did not have distant metastases in the 7th edition, making it impossible to determine the M descriptor in TNM6.

Computation of the stage group does not always require the T or N descriptor. For example, if a patient has distant metastases (M1), the stage group will always be IV. Also, in patients without distant metastases (M0), if the lymph node metastases are classified as N2, any T2 or T3 will yield a stage group of IIIA.

Consequently, in 3,344 of 3,544 records (94.4%), it was possible to definitively determine the stage group. In the remaining 200 patients (5.6%), only a range or a set of stage groups could be identified. Fifty-two patients were either stage IB or IIB, 13 were stage IIB or IIIA, and 85 were stage IIIA or IV. In 20 patients, the stage could range between IA and IIIB. Finally, in 6 patients, the stage could range between IIA and IIIB (Data Supplement). Partial classifications were removed from the ground truth set.

### Evaluating Predictive Network Performance

Predicting 6th edition stage grouping using 7th edition data resulted in an average accuracy of 0.99. Macrosensitivity and microsensitivity were 0.92 and 0.96, respectively. Macrospecificity and microspecificity were 0.99 and 0.99, respectively. Inspection of the confusion matrix showed 2 situations where misclassification was notable. Forty-one percent of the patients who were stage IIB were misclassified as stage IB. Twenty-one percent of the patients classified as stage IIIB were misclassified as stage IB, IIB, or IIIA.

Predicting 7th edition stage grouping using 6th edition data yielded an average accuracy of 0.99. Macrosensitivity and microsensitivity were 0.85 and 0.95, whereas macrospecificity and microspecificity were 0.99 and 0.99, respectively. Again, inspection of the confusion matrix showed 2 notable situations where misclassification was apparent. Stage IIA was misclassified as IA in 53% of patients, and stage IIB was misclassified as IIA or IIIA in 59% of patients. Macro-averaged and micro-averaged sensitivity, specificity, and accuracy for survival are listed in Table 3.

**TABLE 3.**
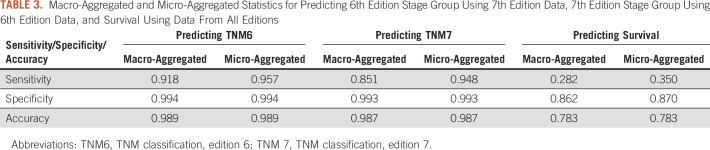
Macro-Aggregated and Micro-Aggregated Statistics for Predicting 6th Edition Stage Group Using 7th Edition Data, 7th Edition Stage Group Using 6th Edition Data, and Survival Using Data From All Editions

**FIG 2.**
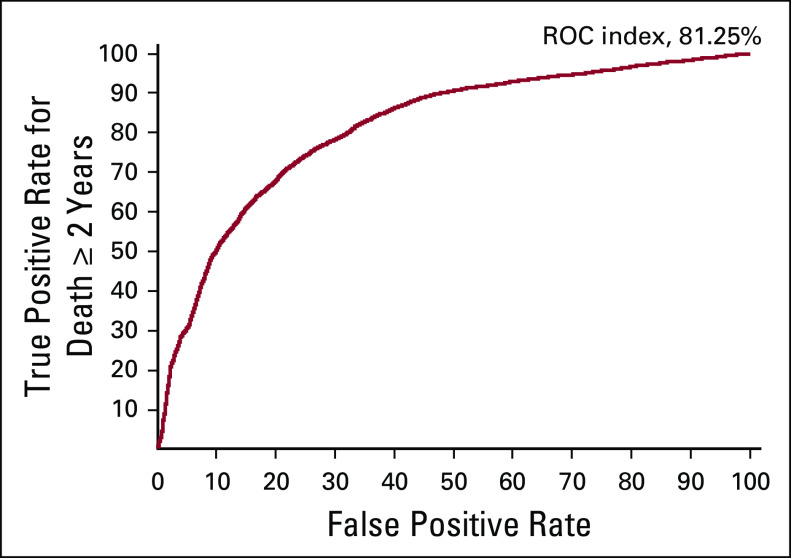
Area under the curve for 2-year survival (0.81), computed using BayesiaLab, and the test set comprising all editions. ROC, receiver operation characteristic.

**FIG 3.**
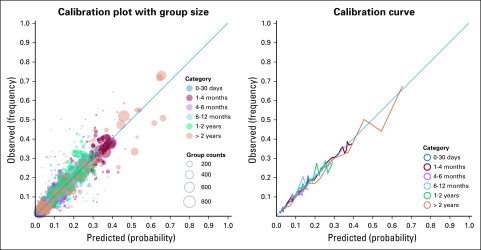
Visualization of model calibration for predicting survival. The calibration (bubble) plot shows, for each subpopulation, the relation between a survival category’s predicted probability (*x*-axis) and its observed frequency in the dataset (*y*-axis). The size of each bubble corresponds to the population’s size in the dataset. The calibration curve shows the same relation, but averages predicted/observed values by applying quantile-based binning with 1,000 samples per bin (21 bins).

The confusion matrices underlying these statistics can be found online (Data Supplement), together with the sensitivity, specificity, and ROC-AUC for predicting each stage group (Data Supplement) and the corresponding set of ROC-AUC curves (Data Supplement). The ROC-AUC for ≥ 2-year survival was 0.81, determined using BayesiaLab, and is shown in Figure 2. Model calibration was computed and is shown in Figure 3.

## DISCUSSION

Mapping class labels requires that each label in the source edition (classification system) can be translated to a label in the target edition. Generally, this is not possible going from coarser to more granular classification systems (eg, from the 6th to the 7th edition of the TNM classification system). In these situations, additional variables are required: either for use *in conjunction with* the original classification or to fully stage a tumor in the target edition. These additional variables are frequently unavailable or obtaining them comes with great cost.

We experienced these issues first hand. When creating the ground truth for evaluating predictive performance, only a relatively small subset of the total test set (12%; patients with a year of diagnosis between 2015 and 2016) could be considered for manual, rule-based reclassification. Even then, reclassification of the T descriptor was not possible in 23% of the patients in the subset. Specifically, we could not determine when a T3 in TNM7 would be a T2 in TNM6 because this required information about the presence of invasion into nearby anatomic structures. Similarly, we could not determine the 6th edition T and M descriptors for 7th edition nonmetastatic, multifocal T4 tumors because this required knowledge of the location(s) of the additional tumor nodules. Therefore, we did not evaluate performance of predicting individual descriptors.

On a more aggregated level, we could not fully determine the 6th edition stage group in 200 patients (5.6%). Although a relatively small number of records was involved, we thought this might still bias the test set. To investigate the potential effect of this bias, we performed an additional analysis. We looked at the (in real-life impossible) worst-case scenario by assuming that all 174 records where we could not decide between 2 stages (eg, stage IB or IIB) would *always* be incorrectly classified, essentially doubling the error. After modifying the confusion matrices in this way, the averaged statistics were recalculated. Micro-averaged and macro-averaged specificity and accuracy were almost unaffected (values changed from 0.99 and 0.99 to 0.98 and 0.96, respectively, that is, a maximum decrease of 0.03). Micro-averaged and macro-averaged sensitivity decreased by a maximum of 0.1.

When using the ground truth set, the BN performed well when reclassifying the TNM stage group between editions: when predicting the 6th edition to the 7th edition data, all aggregated statistics (sensitivity, specificity, and accuracy) were ≥ 0.91. Also, the fact that macro-averaged and micro-averaged statistics were close together implies the model is relatively insensitive to class imbalances. A similar observation can be made for predicting the 7th edition using 6th edition data. The results were only slightly worse, which is to be expected, considering the network has to predict a more granular output from a coarser input. Still, the model had an accuracy of 98%.

Performance for predicting 2-year survival can be considered more than adequate, especially considering the limited number of variables used for making the prediction and the number of possible outcome classes. Adding variables could help, but would come with additional complexity of the model. However, clinical decisions are not just based on the most likely outcome: probability is considered as well. Therefore, binary prediction (eg, 2-year survival: yes/no) seems inadequate to support decision making. Moreover, using a BN in such a way ignores one of its major strengths: the fact that it can communicate (conditional) probabilities. The calibration curves show that the model is well calibrated and that the estimated probabilities are close to the real probabilities.

Inspection of the confusion matrix reveals 4 situations where the model had difficulty in predicting the correct stage group, all related to possible stage shifting. When predicting the 6th edition stage group from 7th edition data, the errors stemmed from difficulty in handling patients where the input (node T_7) is T3. On inspection of the BN, it seemed the model predicted a T3 in the 7th edition to be a T2 with 58% probability and a T3 with 35% probability in the 6th edition. This explains the majority of mistakes made when the predicted descriptor should have been T3. The original priors for this relation were set close to 50-50 (ie, a T3 becoming a T2 or T3 with equal probability) before applying EM learning; therefore, the change in probabilities appears to be an effect of optimizing the relationship between nodes T_567 and death. Additionally, the BN estimates the probability of a T3 in TNM7 being multifocal and thus a T4 in TNM6 to be fairly small, at 4.2%. Even if the actual percentage in the dataset is larger, the value T4 (in TNM6) would never be predicted, because the most probable outcome was selected as prediction.

In predicting the 7th edition stage group from 6th edition data, most of the misclassifications can be explained by the observation that a TNM6 T2 becomes either a T2a, T2b, or T3. Without additional information (ie, tumor diameter), it is not possible to be 100% accurate. Similarly, a T4 in TNM6 can become a T3 or T4, depending on multifocality of the tumor.

Even when using training data with classifications in both the 6th and 7th editions, the BN would not have been able to make any of these distinctions with certainty, because additional information is needed. However, like with predicting survival, the probabilistic reclassification we applied does not need to yield a discrete result: it is possible to assign a probability to each possible outcome, essentially creating a weighted reclassification. This is especially useful when reclassifying large datasets like the NCR.

We conclude that we have successfully created a BN that can aid in determining the TNM stage group of the 6th edition using 7th edition data, and vice versa, by using a training set that does not hold the known classifications in multiple editions but does hold survival to aid in the classification. Knowledge about changes between the editions of the classification system was successfully incorporated by modeling these changes as priors in the CPTs. The model parameters were estimated from data and therefore depend on specific distributions found in the Netherlands. However, considering NSCLC diagnostics, treatment, and survival are comparable in Western/developed countries, we expect the BN can be applied here as is, although validation would be required. This process is likely to work for other tumors and/or editions of the TNM classification system, but additional research is needed to establish generalizability.
